# Emerging Highly Pathogenic Avian Influenza (H5N8) Virus in *Podiceps nigricollis* in Northwest China in 2021

**DOI:** 10.1155/2023/7896376

**Published:** 2023-02-21

**Authors:** Zhen He, Tiezhi Jin, Weikang Wu, Zhe Zhao, Zhenhua Lu, Shuxuan Song, Kun Liu, Zhongjun Shao

**Affiliations:** ^1^Department of Epidemiology, School of Public Health, Air Force Medical University, Xi'an 710032, China; ^2^Shaanxi Institute of Zoology, Xi'an 710032, China; ^3^Gansu University of Chinese Medicine, Lanzhou 730000, China

## Abstract

Three highly pathogenic avian influenza (H5N8) viruses were detected in the migratory bird *Podiceps nigricollis* in Northwest China in June 2021. Phylogenetic analysis indicated that these H5N8 isolates belonged to clade 2.3.4.4b, which were highly homologous to strains isolated in China and South Korea. In this study, H5N8 virus infection in *Podiceps nigricollis* was detected using Oxford Nanopore Technologies sequencing technology and caused pathological changes in multiple organs.

## 1. Introduction

Avian influenza virus (AIV) is a member of the influenza A virus of the Orthomyxoviridae family and can cause avian disease, a public health threat. In recent years, AIVs, particularly the highly pathogenic AIV (HPAIV) A (H5N8), have caused numerous outbreaks worldwide in both poultry and wild birds, especially in autumn and winter. Migratory birds such as waterfowl are natural hosts of AIV. The long-distance spread of HPAIV is closely associated with wild bird migration, particularly H5N8 and H5N1 [1, 2].

H5N8 HPAIV of branch 2.3.4 was first detected in Chinese poultry in 2010 [3], although it did not cause a large-scale outbreak. In 2014, multiple H5N8 HPAI outbreaks were reported in South Korea, China, Japan, Europe, and North America, triggering the first intercontinental epidemic [4, 5]. By 2016, H5N8 HPAIV spread to Europe, the Middle East, Africa, and other countries and regions, leading to a second global epidemic [6]. More importantly, several H5Nx AIVs have a zoonotic potential and cross the species barrier from birds to humans, confirmed by the first H5N8 HPAIV infection in humans in December 2020. H5N8 HPAIV has become one of the important biosecurity issues threatening the world; thus, the zoonotic potential of AIVs warrants continuous, vigilant monitoring to avert further spread and disastrous pandemics.

## 2. Materials and Methods

From May 27 to June 10, 2021, dead or seriously sick birds were found in the Hongjiannao Nature Reserve, Shenmu County, Yulin City, Shaanxi, Northwest China. 14 migratory birds were collected, including 13 black-necked grebes (*Podiceps nigricollis*) and one great crested grebe (*Podiceps cristatus*) in the Hongjiannao Nature Reserve (latitude: 39.071876°N, longitude: 109.929433°E) from June 2 to 6, 2021. Some organs of the 14 dead birds were dissected and collected by the Shaanxi Institute of Zoology (Northwest Institute of Endangered Zoological Species). These samples were then sent to our laboratory.

The lung, liver, heart, trachea, and kidney tissues from dead migratory birds were ground, and a sterile 0.45 *μ*m polyvinylidene fluoride (PVDF) membrane (Millipore, USA) was used to remove eukaryotic and bacterial cell-sized particles. The viral nucleic acids (RNA) were extracted using the QIAamp MinElute Virus Spin Kit (Qiagen) [7]. Viral double-strand cDNA was synthesized with reverse transcriptase MLV and specific primer RRM (5′-GACCATCTAGCGACCTCCAC-NNNNNN-3′). The final amplification reaction was performed with the double-strand cDNA template and specific primer (5′-GCCGGA‐GCTCTGCAGAATTC-3′). GridION library preparation was performed according to the manufacturer's instructions for barcoding cDNA/DNA and native DNA (SQK-LSK109 and EXP-NBD104). Oxford Nanopore Technologies MinKNOW software was used to collect raw sequence data. GridION sequencing was run for up to 26 h. The Guppy base-calling tool was used to trim adaptor and barcode sequences for demultiplexing ONT reads from FAST5 files. The filtered reads were assembled and blasted with the avian influenza virus (AIV) database to search for the best-matched reference sequences. Subsequently, the filtered reads were mapped to the reference sequences using Minimap2 and converted with SAM tools [8, 9].

Meanwhile, we downloaded the AIV sequences from the GenBank and GISAID databases to create a local AIV database (updated December 31, 2021). Then, using each sequence of the eight H5N8 viruses as a query, Blastn was performed with default parameters against the local AIV database. Subsequently, the first 100 gene sequences in the output were collected. Thus, eight datasets corresponding to the eight AIV sequences were obtained. Subsequently, the optimal model and the maximum likelihood phylogenetic trees were constructed by Mega-X. After selecting the optimal model calculated before, the phylogenetic tree was constructed using the maximum likelihood method, and the bootstrap value was set to 1000.

In addition, the lung, liver, heart, trachea, and kidneys were collected from dead migratory birds. These tissues were fixed in 4% formaldehyde (neutral buffered) and embedded in paraffin. Hematoxylin-eosin staining was performed on sections of these tissues to observe the histopathological changes.

## 3. Results and Discussion

On May 27, 2021, 4,249 dead wildfowl, primarily *Podiceps nigricollis*, were found in the Hongjiannao Nature Reserve, Shenmu, Shaanxi. These infected birds had visible clinical features such as weakness and dyspnea. Oropharyngeal swabs and organs from birds were collected for pathogen identification. Before the laboratory confirmation of the pathogen, the Ministry of Agriculture and Rural Affairs implemented prompt and strict control measures, including the harmless treatment of all dead and infected wildfowl, demarcating epidemic areas, strictly controlling vehicles and people entering and leaving the area, and disinfecting the lake area contaminating with the virus and reduce the risk of human infection.

**Figure 1:**
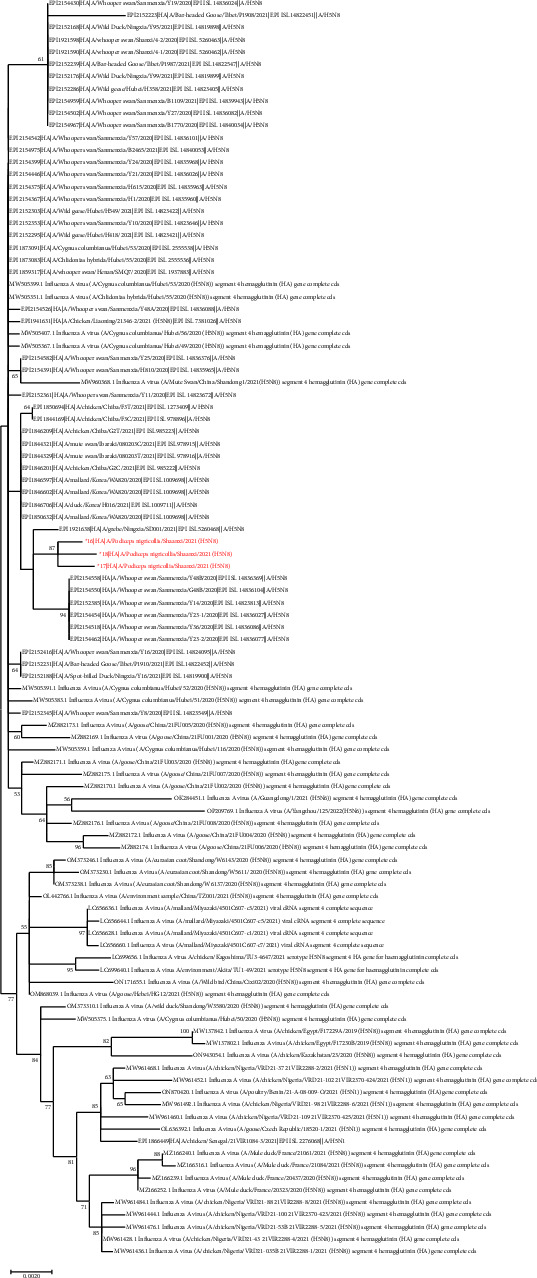
Maximum likelihood phylogenetic trees of eight sequences of the H5N8 viruses. Maximum likelihood phylogenetic tree of the hemagglutinin (HA) sequence. 16 : 16/A/*Podiceps nigricollis*/Shaanxi/2021(H5N8); 17 : 17/A/*Podiceps nigricollis*/Shaanxi/2021(H5N8); 18 : 18/A/*Podiceps nigricollis*/Shaanxi/2021(H5N8).

**Figure 2:**
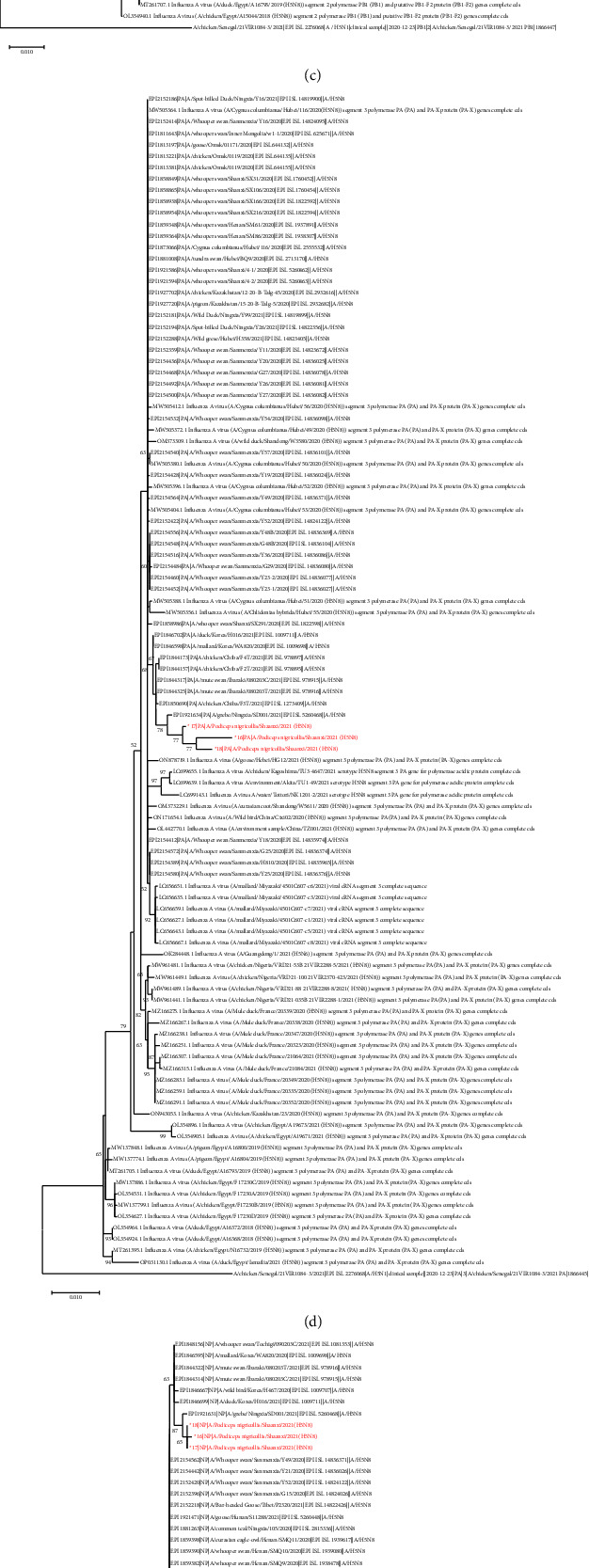
Maximum likelihood phylogenetic trees of the eight sequences of the H5N8 viruses. Maximum likelihood phylogenetic tree of the (a) neuraminidase (NA) sequence, (b) polymerase basic (PB2) sequence, (c) polymerase basic (PB1) sequence, (d) polymerase (PA) sequence, (e) nucleoprotein (NP) sequence, (f) matrix protein (MP) sequence, and (g) nonstructural protein (NS) sequence. H5N8 viruses from Shaanxi isolated in 2021 are marked in red. Maximum likelihood phylogenetic trees were constructed using MEGA-X software. 16 : 16/A/*Podiceps nigricollis*/Shaanxi/2021(H5N8); 17 : 17/A/*Podiceps nigricollis*/Shaanxi/2021(H5N8); 18 : 18/A/*Podiceps nigricollis*/Shaanxi/2021(H5N8).

Total viral RNA was extracted from organ samples and assessed for nucleic acid concentration and purity to confirm the pathogen. Full-length H5N8 virus genome sequences of three strains were obtained using direct RNA sequencing (i.e., Oxford Nanopore Technologies sequencing). All eight segment sequences of the three strains were blasted against the NCBI database, and the highest homology with the eight segments was the strain found in China and Korea in 2021 (Table 1). The ML-tree of hemagglutinin (HA) is shown in Figure 1, while the other fragment sequences are shown in Figure 2. The ML-tree of the hemagglutinin (HA) sequence showed that the three H5N8 strains had the highest sequence homology with H5N8 strains isolated from poultry and wild birds in Korea (i.e., 99.5%; Table 1), all of which belonged to the 2.3.4.4b clade. In October 2020, the H5N8 virus was detected in swans in Inner Mongolia, China [10].

Meanwhile, similar viruses were reported in Shandong, China, in late November 2020 [11]. According to the reports, birds can pass through China through three bird migratory routes: the East Asian-Australasian flyway, the Central Asian flyway, and the Black Sea-Mediterranean flyway. Several migratory flyways in Eurasia have overlapping breeding areas. However, the most common bird migration pattern is flying north to breed in spring and summer and returning to warmer regions in autumn [12]. Swans spend the winter in Southern China and Southeast Asia and breed in Mongolia or East Asia (such as South Korea and Japan) along the East Asian-Australasian flyway. Migratory birds such as cardinals spend the winter in South Asia (i.e., India, Nepal, Bangladesh, and Myanmar) and breed along the Central Asian flyway in Qinghai or Mongolia [13]. Shaanxi (including Yulin City) is a key stopover site during the migration of wild birds in China, and the Hongjiannao Nature Reserve is an ideal habitat for migratory birds, especially waterfowl. Thus, H5N8 HPAIV detected in *P. nigricollis* was likely to be transmitted during the migration and reproduction of migratory birds. Combining the phylogenetic analysis of the virus with the migration pattern of wild birds, these birds likely spent the winter in Southern China in the fall and winter of 2020 and then bred along the East Asian-Australasian flyway to spread the virus to Northwest China.

**Figure 3:**
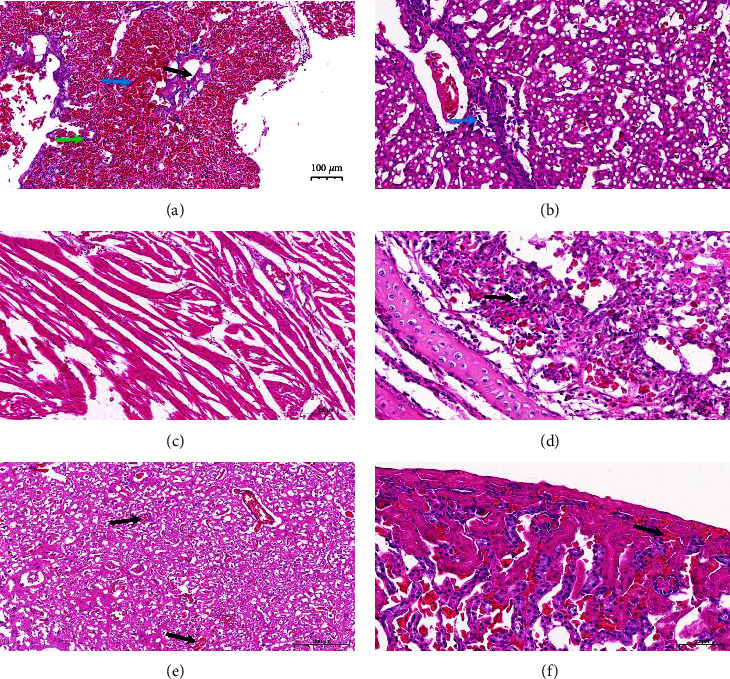
Pathological changes in the lungs, liver, heart, trachea, brain, and kidney of dead migratory birds infected with H5N8. (a) Pathological changes in the lung. Inflammatory cell infiltration (green arrow), focal necrosis (black arrow), and erythrocyte (blue arrow). (b) Pathological changes in the liver, inflammatory cells (blue arrow) near the portal area, and severe steatosis. (c) No obvious pathological changes in the heart. (d) Pathological changes in the trachea, hypertrophy of tracheal mucosa, and infiltration of inflammatory cells in stroma (black arrows). (e) Pathological changes in the brain and different degrees of cerebral parenchymal vascular congestion (black arrows). (f) Pathological changes in the kidney. Interstitial hemorrhage (black arrows) was obvious.

To further explore the pathogenicity of the H5N8 virus in migratory birds, tissues of three dead migratory birds were stained (Figure 3). Histopathological analysis showed inflammatory cell infiltration and necrosis in these organs. In detail, severe bleeding in the lungs and numerous erythrocytes in the respiratory lumen, accompanied by necrotic foci, were found surrounded by inflammatory cell infiltration. Inflammatory cells near the portal area, and severe steatosis were observed in the liver. The mucosal layer of the trachea was hypertrophic, with inflammatory cell infiltration in the stroma. Blood vessels of the cerebral parenchyma were congested to different degrees. The renal interstitium was congested with obvious inflammatory cell infiltration. However, no obvious pathological changes were observed in the heart. According to the histopathological analysis, the pathological changes in the lungs, liver, trachea, brain, and kidney were highly pathogenic, consistent with related HPAIV reports. However, whether severe liver steatosis is associated with H5N8 needs further research. Interestingly, we found that the sequence obtained in this study was highly homologous with the sequence (A/grebe/Shaanxi/SD001/2021 (A/H5N8), A/grebe/Ningxia/SD001/2021 (A/H5N8)) uploaded by Professor Cui of Harbin Veterinary Research Institute in the GISAID database, indicating that it is feasible and effective to combine the filtering extraction method with the Oxford Nanopore technology.

In conclusion, H5N8 HPAIV was detected during the spring migration of *P. nigricollis* in Shaanxi, China, and phylogenetic analysis illustrated the pathways of the introduction of the H5N8 virus to Central China. This finding warrants the need to strengthen the monitoring of various migratory waterfowl at the main stopover sites and breeding habitats to prevent the development and spread of HPAIV.

## Figures and Tables

**Table 1:** Highest nucleotide identity of the eight H5N8 virus sequences with the sequences from the NCBI and GISAID.

Genes	Viruses with the highest nucleotide identity	Accession number	Identity	Homology (%)
PB2	A/grebe/Ningxia/SD001/2021 (A/H5N8)	EPI1921635	16 : 2149/2161	99.44–99.78
			17 : 2273/2280	
			18 : 2275/2280	

PB1	A/chicken/Chiba/F3T/2021 (A/H5N8)	EPI1850692	16 : 2271/2277	99.60–99.73
			17 : 2268/2277	
			18 : 2269/2277	

PA	A/grebe/Ningxia/SD001/2021 (A/H5N8)	EPI1921634	16 : 2122/2151	98.65–99.48
			17 : 2140/2151	
			18 : 2131/2152	

HA	A/mallard/Korea/WA820/2020 (A/H5N8)	EPI1850632	16 : 1699/1704	99.59–99.70
			17 : 1698/1704	
			18 : 1492/1498	

NP	A/grebe/Ningxia/SD001/2021 (A/H5N8)	EPI1921631	16 : 1494/1497	99.86–99.79
			17 : 1495/1497	
			18 : 1495/1497	

NA	A/grebe/Ningxia/SD001/2021 (A/H5N8)	EPI1921637	16 : 1406/1413	99.50–99.78
			17 : 1410/1413	
			18 : 1409/1413	

MP	A/grebe/Ningxia/SD001/2021 (A/H5N8)	EPI1921633	16 : 978/982	99.59–99.69
			17 : 979/982	
			18 : 979/982	

NS	A/grebe/Ningxia/SD001/2021 (A/H5N8)	EPI1921632	16 : 837/838	99.88–100
			17 : 837/838	
			18 : 838/838	

*Note*. 16 : 16/A/*Podiceps nigricollis*/Shaanxi/2021(H5N8); 17 : 17/A/*Podiceps nigricollis*/Shaanxi/2021(H5N8); 18 : 18/A/*Podiceps nigricollis*/Shaanxi/2021(H5N8).

## Data Availability

The sequencing data used to support the findings of this study are available from the corresponding author upon request.
